# Effect of obesity status on adolescent bone mineral density and saturation effect: A cross-sectional study

**DOI:** 10.3389/fendo.2022.994406

**Published:** 2022-10-14

**Authors:** Gao-Xiang Wang, Ze-Bin Fang, Hui-Lin Li, De-Liang Liu, Shu-Fang Chu, Heng-Xia Zhao

**Affiliations:** ^1^ Department of Endocrinology, Shenzhen Traditional Chinese Medicine Hospital Affiliated to Nanjing University of Chinese Medicine, Shenzhen, China; ^2^ Department of Endocrinology, The Fourth Clinical Medical College of Guangzhou University of Chinese Medicine, Shenzhen, China; ^3^ Department of Endocrinology, Shenzhen Traditional Chinese Medicine Hospital, Shenzhen, China

**Keywords:** Bone mineral density, bone mineral content, body mass index, waist circumference, Fat/lean mass, NHANES, osteoporosis, Adolescents

## Abstract

**Background:**

The effect of obesity status on bone mineral density (BMD) in adolescents and whether there is a saturation effect is still insufficient. A cross-sectional study of adolescents aged 12–19 was conducted to investigate them.

**Methods:**

Weighted multivariate linear regression models were used to assess the relationship between obesity status and BMD *via* datasets from the National Health and Nutrition Examination Survey 2011–2018. The nonlinear relationships and saturation values were ascertained by fitting smooth curves and analyzing saturation effects. At the same time, the subgroup stratified analysis was also performed.

**Results:**

4056 adolescents were included in this study. We found that body mass index (BMI) and waist circumference (WC) were significantly associated with total BMD, which remained significant in subgroups stratified by age, gender, standing height, and ethnicity. We also noticed an inverse correlation between left leg fat/lean mass and left leg BMD, which was only significant in males and other races. Fitting smooth curve and saturation effect analysis showed that BMI, WC, left leg fat/lean mass, and BMD had a specific saturation effect. There was a saturation effect on bone mineral density in adolescents with a BMI of 22 kg/m^2^, a WC of 70.5 cm, or a left leg fat/lean mass of 0.2994.

**Conclusions:**

We found a positive saturation effect of BMI and WC with BMD and a negative saturation effect of left leg fat/lean mass with BMD. Appropriate obesity status allows adolescents to have better bone mass development but not excessive obesity.

## Introduction

Osteoporosis (OP) is a degenerative disease of the bones that results in weakened bones, weakened microarchitecture, increased fragility, and increased fracture risk (1). According to an epidemiological survey, at least 200 million people globally suffer from OP, which is predicted to rise substantially over time (2). A new study predicts that more than 70 million more people in the United States will be diagnosed with OP, or bone loss, by 2030 (3). OP fractures will not only have a terrible psychological influence on the patient, but also place a significant financial strain on the entire family (4, 5). Bone mineral density (BMD) is one of the most critical diagnostic markers of OP, and obesity status is closely related to BMD. Both Ma et al. (6). and Y et al. (7). found a positive saturation effect between obesity status and BMD in people older than 50.

Adolescence is the most critical period to reach peak BMD (8). To our knowledge, however, existing studies examining the effects of obese status on adolescent BMD and the existence of a saturation effect are insufficient and contentious. Although Yujuan et al. (9). concluded that Body mass index (BMI) was positively associated with BMD in adolescents, their study did not adjust for some factors that have been shown to affect BMD in adolescents, such as regulating serum creatinine (10) and uric acid (11), and only considered BMI and did not consider indicators of other obesity conditions, such as waist circumference (WC) and body fat mass. Kátia et al. (12). found that obesity negatively impacts skeletal development in adolescents, leading to underdevelopment of bone mass. In a cross-sectional study, Yin et al. (13). found a negative correlation between WC and lumbar BMD among people aged 8 to 18. A survey of 982 Korean young people aged 12–19 found a negative relationship between body fat mass and total-body-less-head BMD in males (14). A study of 795 adolescent participants by Hee-Cheol et al. (15). found no association between body fat mass and BMD after adjusting for lean body mass.

In this study, we used the NHANES database to conduct a cross-sectional analysis to explore the effect of several indicators of obesity status (BMI, WC, and fat/lean mass) on adolescent BMD and whether saturation effects exist. This study’s results can be used as a guide for therapy, which can allow adolescents to have better bone mass development but not excessive obesity.

## Methods

### Data source and study population

Our cross-sectional analysis was supported by data from the NHANES 2011–2018. The survey is aimed at patients from all backgrounds of life in America. All of the subjects were subjected to a battery of tests, consisting of BMI, WC, Standing height, lab tests, and standardized questionnaires concerning their age, gender, race/ethnicity, moderate activity, and household income-to-poverty ratio. This data was utilized in the evaluation of the prevalence and severity of a wide variety of diseases, as well as in the formulation of public health policies and the provision of medical care.

**Figure 1:**
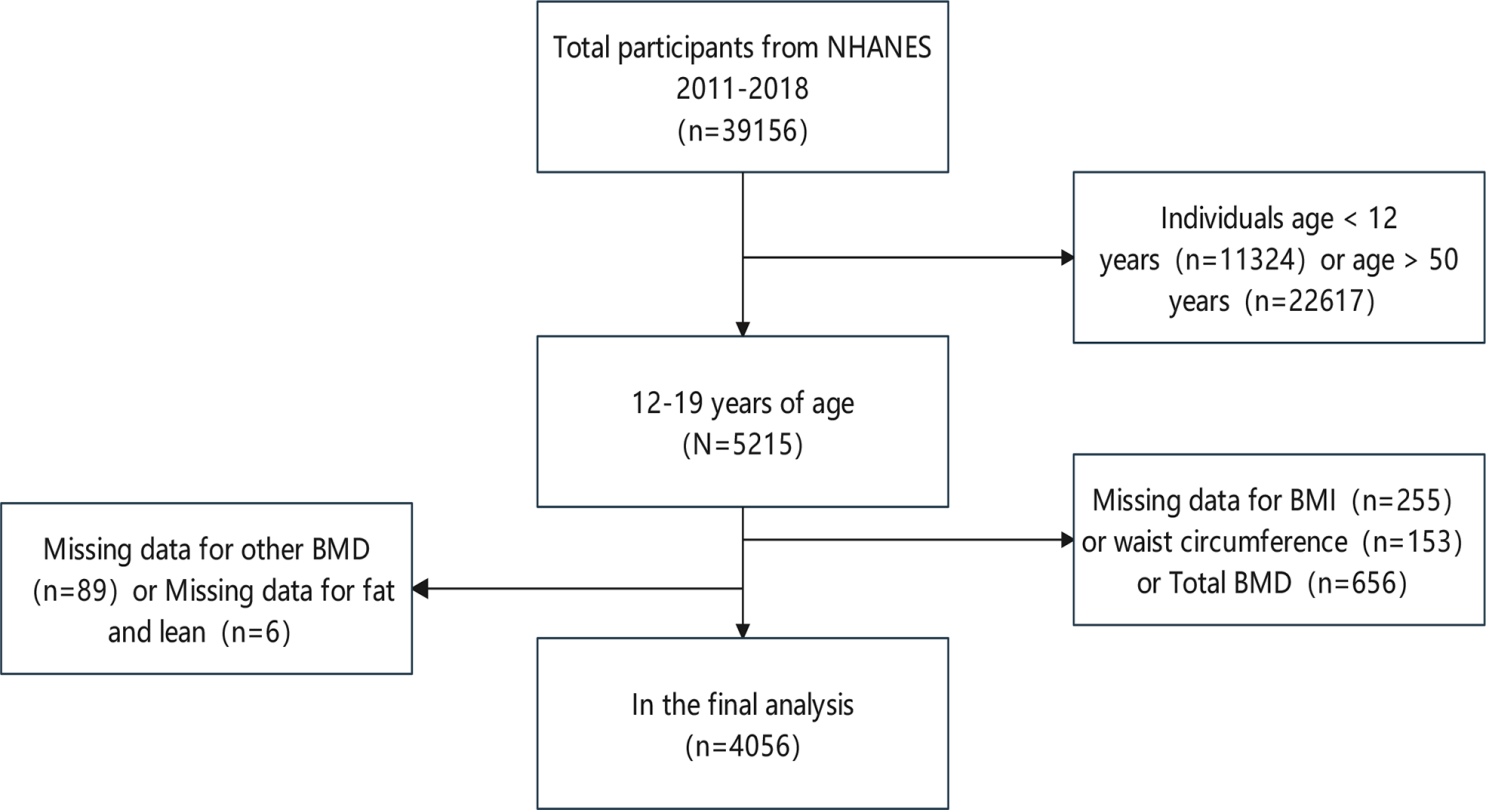
Flowchart of participant selection.

The participants in the study ranged in age from 12 to 19. From a total of 39,156 participants between 2011 and 2018, we excluded 11,324 children under the age of 12 and 22,617 adults over the age of 19; 255 subjects with missing BMI information; 153 subjects with missing WC information; 656 subjects with missing total BMD information, as well as 89 subjects with missing other BMD; and 6 subjects with missing fat and lean. Following the aforesaid screening, data from 4056 participants was included (**Figure 1**).

### Ethics statement

The NHANES required every individual who took part in the survey to sign an informed consent form, which was then reviewed and authorized by the National Center for Health Statistics Ethics Review Board. The data can now be accessed by the general public following privacy-preserving. It is already possible to transform data into a form that can be analyzed. All statistics would be used for data analysis and all studies will be done in compliance with applicable laws and standards provided we comply with the study’s data usage guidelines.

### Covariates

Self-reporting of completed questions included information on age, gender, race/ethnicity, moderate activity, and the percentage of household income in poverty. Professional physical examination using conventional procedures, including assessment of weight, height, and WC. The height and weight of the respondents were measured by first reminding them to take off their shoes and any heavy clothing. Afterward, their BMI was measured by dividing their total body weight by the square of their height. Drawing the right midaxillary line by drawing a horizontal line above the highest lateral border of the right ilium, and positioning a tape measure at the intersection of the two lines are all necessary steps in the process of measuring the WC. During the process of the research project, measurements were taken of things like serum alkaline phosphatase, serum calcium, serum phosphorus, uric acid, total cholesterol, triglyceride, blood urea nitrogen, serum creatinine, and urine albumin-to-creatinine ratios. This took place at the scheduled visit.

### Outcome variable

Whole-body scans using dual-energy X-ray absorptiometry (DXA) were performed on all of the subjects. To calculate the BMC and BMD, a qualified and certified radiographer used a QDR-4500A fan-beam densitometer, DXA images from Hologic, Inc. in Bedford, Massachusetts, and Hologic APEX (version 4.0) software. For more information on how to collect covariate data and how to measure WC, BMI, fat, lean, BMC, and BMD, among other things, go to www.cdc.gov/nchs/nhanes/.

### Statistical analysis

We used EmpowerStats (http://www.empowerstats.com) and R (3.4.4 version) software for statistical analysis. This study conducted these analyses to see whether categorical and continuous variables differed significantly. This was accomplished through the utilization of multivariate linear regression models to calculate the β value and 95% confidence interval (CI). According to the Strengthening the Reporting of Observational Studies in Epidemiology guidelines, all covariates were adjusted in all models.

## Result

### Characteristics of participants

The weighted distribution of the basic information of the population in this investigation is shown in **Table 1**. The number of individuals was 2151 males and 1905 females. There were no highly relevant differences between male and female participants in terms of age, the ratio of household income to poverty, WC, pelvis BMD, and thoracic BMC. Moderate activity, alkaline phosphatase, serum calcium, serum phosphorus, serum uric acid, triglycerides, blood urea nitrogen, serum creatinine, standing height, total BMD, left leg BMD, left arm BMD, trunk BMD, left rib BMD, total BMC, lumbar spine BMC, left leg BMC, left arm BMC, left rib BMC, pelvis BMC, trunk BMC baseline in male participants were higher than female in terms of bone mineral resources and lower than females in terms of total cholesterol, urinary albumin creatinine ratio, BMI, WC, total fat lean, trunk fat lean, left leg fat lean, left arm fat lean, lumbar spine BMD, thoracic BMD, head BMD, and head BMC, and these differences were statistically significant.

**Table 1:** Weighted characteristics of the study sample.

	Male (n = 2151)	Female (n = 1905)	*P* value
**Age (years)**	15.36 ± 2.26	15.40 ± 2.25	0.527
**Race/ethnicity (%)**			0.046
Mexican American	436 (20.27%)	412 (21.63%)	
Other Hispanic	201 (9.34%)	225 (11.81%)	
Non-Hispanic White	590 (27.43%)	497 (26.09%)	
Non-Hispanic Black	533 (24.78%)	428 (22.47%)	
Other race - including multi-racia	391 (18.18%)	343 (18.01%)	
**Ratio of family income to poverty (%)**	2.08 ± 1.45	2.02 ± 1.48	0.221
**Moderate activities (%)**			0.003
No	632 (29.38%)	655 (34.38%)	
Yes	1167 (54.25%)	955 (50.13%)	
No record	352 (16.36%)	295 (15.49%)	
**Alkaline phosphatase (u/L)**	168.72 ± 102.53	102.28 ± 56.09	<0.001
**Serum calcium (mmol/L)**	2.41 ± 0.07	2.38 ± 0.07	<0.001
**Serum phosphorus (mmol/L)**	1.43 ± 0.22	1.36 ± 0.18	<0.001
**Serum uric acid (umol/L)**	329.98 ± 67.62	266.78 ± 55.46	<0.001
**Total cholesterol (mmol/L)**	3.97 ± 0.71	4.11 ± 0.74	<0.001
**Triglyceride (mmol/L)**	1.13 ± 0.79	1.03 ± 0.62	<0.001
**Blood urea nitrogen (mmol/L)**	4.18 ± 1.18	3.71 ± 1.06	<0.001
**Serum creatinine (umol/L)**	68.54 ± 14.92	58.16 ± 11.24	<0.001
**Urinary albumin creatinine ratio (mg/g)**	19.14 ± 98.89	34.80 ± 138.16	<0.001
**Standing height (cm)**	169.23 ± 9.37	159.95 ± 6.87	<0.001
**Body mass index (kg/m^2^)**	23.84 ± 6.01	24.34 ± 6.13	0.009
**Waist circumference (cm)**	82.00 ± 15.52	81.71 ± 14.30	0.532
**Total fat/lean mass**	0.36 ± 0.16	0.57 ± 0.17	<0.001
**Trunk fat/lean mass**	0.30 ± 0.16	0.48 ± 0.18	<0.001
**Left Leg fat/lean mass**	0.44 ± 0.20	0.73 ± 0.19	<0.001
**Left Arm fat/lean mass**	0.38 ± 0.22	0.70 ± 0.25	<0.001
**Head fat/lean mass**	0.36 ± 0.01	0.36 ± 0.01	<0.001
**Total bone mineral density (g/cm^2^)**	1.05 ± 0.13	1.02 ± 0.10	<0.001
**Lumbar Spine Bone Mineral Density (g/cm^2^)**	0.94 ± 0.17	1.00 ± 0.14	<0.001
**Left Leg Bone Mineral Density (g/cm^2^)**	1.14 ± 0.16	1.07 ± 0.12	<0.001
**Left Arm Bone Mineral Density (g/cm^2^)**	0.74 ± 0.10	0.67 ± 0.06	<0.001
**Trunk Bone Mineral Density (g/cm^2^)**	0.87 ± 0.14	0.85 ± 0.10	<0.001
**Pelvis Bone Mineral Density (g/cm^2^)**	1.18 ± 0.20	1.18 ± 0.16	0.404
**Thoracic Bone Mineral Density (g/cm^2^)**	0.74 ± 0.12	0.75 ± 0.10	<0.001
**Head Bone Mineral Density (g/cm^2^)**	1.75 ± 0.32	1.92 ± 0.34	<0.001
**Left Rib Bone Mineral Density (g/cm^2^)**	0.64 ± 0.10	0.61 ± 0.07	<0.001
**Total Bone Mineral Content (g)**	2173.59 ± 530.31	1897.75 ± 355.95	<0.001
**Lumbar Spine Bone Mineral Content (g)**	50.82 ± 15.92	48.47 ± 11.00	<0.001
**Left Leg Bone Mineral Content (g)**	431.73 ± 107.02	350.36 ± 70.20	<0.001
**Head Bone Mineral Content (g)**	415.73 ± 87.97	424.25 ± 85.83	0.002
**Left Arm Bone Mineral Content (g)**	157.50 ± 48.72	127.14 ± 28.49	<0.001
**Left Rib Bone Mineral Content (g)**	80.15 ± 21.00	70.87 ± 15.59	<0.001
**Thoracic Bone Mineral Content (g)**	94.78 ± 28.37	94.05 ± 21.42	0.36
**Pelvis Bone Mineral Content (g)**	263.45 ± 91.20	223.62 ± 58.35	<0.001
**Trunk Bone Mineral Content (g)**	566.30 ± 165.39	506.35 ± 109.18	<0.001

Continuous variables are presented as Mean ± SD, P-value was calculated by a weighted linear regression model. Categorical variables are presented as %, P-value was calculated by chi-square test.

### The association between BMI and BMD

**Figure 2:**
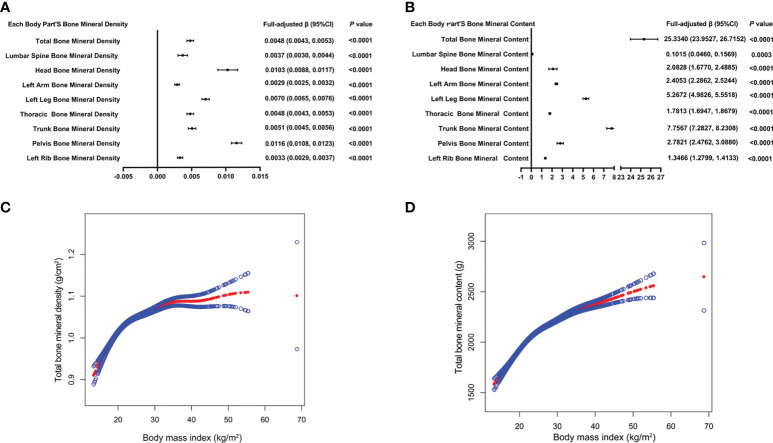
The forest plots of each body part’s bone mineral density or bone mineral content and body mass index, respectively **(A, B)**. Association of total bone mineral density and bone mineral content with body mass index **(C, D)**. The solid red line represents the smooth curve fit between variables. Blue bands represent the 95% confidence interval from the fit. All confounding factors were adjusted.

**Table 2** presents three distinct weighted multiple linear regression models. All variables were adjusted, and there was a statistically significant positive correlation between BMD and BMI in all three models. When stratifying BMI by quartile and using the lowest quartile as a reference point, the trend analysis was statistically significant *(P* for trend < 0.001). In subgroups stratified by gender, age, standing height, and race, the positive associations for total BMD, left leg BMD, and BMI remained significant. Notably, in the age-stratified subgroup, this association of BMI with lumbar spine BMD was not observed among adolescents aged 12, 13, and 17 - 19 years. **Figures 2A, B** are the forest plots of each body part’s BMD or BMC and BMI, respectively, and each body part’s BMD or BMC and BMI has significant correlations. **Figures 2C, D** are the smooth curve fitting graphs of total BMD or total BMC and BMI, respectively. When we smooth curve fit the revised model, there is a saturating effect for total BMD and BMI (**Figure 2C**). We conducted a saturation effect model analysis to determine the BMI tipping point and determined that the BMI saturation effect value was 22 kg/m^2^. When BMI < 22 kg/m^2^, BMD increased by 0.0136 g/m^2^ for per unit increase in BMI; for BMI > 22 kg/m^2^, BMD increased by 0.0027 g/m^2^. In addition, when stratified by age, we discovered that the BMI of teenagers at each age had a saturation effect, as shown in **Table 3**. Likewise, when we separated the data by gender, we found that both males and females had BMI saturation values.

**Table 2:** Association between body mass index (kg/m^2^) and bone mineral density (g/cm^2^).

Exposure	Total BMDβ (95% CI)	Lumbar Spine BMD β (95% CI)	Left Leg BMD β (95% CI)
Quintiles of body mass index (kg/m2)			
< 18.5	reference	reference	reference
>= 18.5, < 25	0.0493 (0.0416, 0.0569)	0.0577 (0.0471, 0.0683)	0.0645 (0.0555, 0.0735)
>= 25, < 30	0.0708 (0.0615, 0.0801)	0.0663 (0.0535, 0.0792)	0.0984 (0.0875, 0.1094)
>= 30	0.1012 (0.0906, 0.1117)	0.0815 (0.0669, 0.0961)	0.1450 (0.1326, 0.1574)
P for trend	< 0.001	< 0.001	< 0.001
**Stratified by gender**			
Male	0.0048 (0.0040, 0.0055)	0.0044 (0.0035, 0.0054)	0.0065 (0.0056, 0.0074)
Female	0.0048 (0.0041, 0.0055)	0.0031 (0.0021, 0.0041)	0.0078 (0.0071, 0.0086)
**Stratified by age (years old)**			
12	0.0031 (0.0019, 0.0043)	-0.0412 (-0.1757, 0.0932)	0.0069 (0.0055, 0.0083)
13	0.0045 (0.0033, 0.0056)	-0.0463 (-0.1734, 0.0808)	0.0067 (0.0053, 0.0082)
14	0.0049 (0.0035, 0.0063)	0.1571 (0.0141, 0.3001)	0.0068 (0.0053, 0.0084)
15	0.0058 (0.0044, 0.0072)	0.2323 (0.0737, 0.3910)	0.0074 (0.0058, 0.0090)
16	0.0050 (0.0037, 0.0063)	0.1580 (0.0142, 0.3018)	0.0076 (0.0061, 0.0091)
17	0.0049 (0.0036, 0.0063)	0.1060 (-0.0453, 0.2573)	0.0073 (0.0057, 0.0088)
18	0.0023 (0.0012, 0.0035)	0.0639 (-0.0710, 0.1987)	0.0049 (0.0036, 0.0062)
19	0.0032 (0.0018, 0.0046)	-0.0447 (-0.1906, 0.1013)	0.0049 (0.0033, 0.0066)
**Stratified by standing height (cm)**			
Q1 (132.9-160.3)	0.0056 (0.0048, 0.0064)	0.0042 (0.0030, 0.0053)	0.0094 (0.0084, 0.0103)
Q2 (160.4-169)	0.0043 (0.0034, 0.0052)	0.0029 (0.0017, 0.0041)	0.0065 (0.0054, 0.0076)
Q3 (169.1-190.9)	0.0047 (0.0038, 0.0056)	0.0044 (0.0031, 0.0056)	0.0061 (0.0050, 0.0072)
**Stratified by race**			
Mexican American	0.0044 (0.0033, 0.0055)	0.0031 (0.0017, 0.0045)	0.0068 (0.0055, 0.0081)
Other Hispanic	0.0060 (0.0045, 0.0076)	0.0066 (0.0044, 0.0088)	0.0088 (0.0069, 0.0106)
Non-Hispanic White	0.0050 (0.0040, 0.0060)	0.0034 (0.0021, 0.0048)	0.0075 (0.0064, 0.0087)
Non-Hispanic Black	0.0037 (0.0027, 0.0047)	0.0039 (0.0025, 0.0053)	0.0057 (0.0046, 0.0069)
Other race	0.0052 (0.0040, 0.0064)	0.0042 (0.0025, 0.0059)	0.0065 (0.0051, 0.0079)

Adjusted for all confounding factors (age, gender, standing height, race, ratio of family income to poverty, moderate activities, alkaline phosphatase, serum calcium, serum phosphorus, serum uric acid, total cholesterol, triglyceride, blood urea nitrogen, serum creatinine, urinary albumin creatinine ratio).

The model is not adjusted for the stratification variable itself in the subgroup analysis.

**Table 3:** Saturation effect analysis of obesity status and bone mineral density (g/cm^2^).

Bone mineral density (g/cm^2^)	Model: saturation effect analysis		
	Body mass index(kg/m^2^)	Waist circumference (cm)	Left leg fat/lean mass
**Turn point**	**22**	**70.5**	**0.2994**
< Turn point, effect1	0.0136 (0.0120, 0.0152)	0.0054 (0.0041, 0.0067)	-0.3185 (-0.4569, -0.1802)
> Turn point, effect2	0.0027 (0.0021, 0.0033)	0.0010 (0.0007, 0.0012)	-0.0096 (-0.0282, 0.0091)
**Stratified by gender**			
**Turn point of males**	**22**	**78**	**0.2893**
< Turn point, effect1	0.0135 (0.0113, 0.0158)	0.0032 (0.0023, 0.0042)	-0.2582 (-0.4292, -0.0872)
> Turn point, effect2	0.0026 (0.0017, 0.0035)	0.0005 (0.0002, 0.0009)	-0.0341 (-0.0631, -0.0051)
**Turn point of females**	**21.5**	**102**	**0.8016**
< Turn point, effect1	0.0141 (0.0116, 0.0166)	0.0020 (0.0016, 0.0024)	-0.0049 (-0.0440, 0.0343)
>Turn point, effect2	0.0031 (0.0024, 0.0039)	-0.0001 (-0.0009, 0.0008)	0.0482 (-0.0007, 0.0971)
**Stratified by age**			
**Turn point of 12 years old**	**17.6**	**64.5**	**0.3082**
< Turn point, effect1	0.0222 (0.0130, 0.0314)	0.0053 (0.0014, 0.0093)	-0.4383 (-1.0353, 0.1588)
>Turn point, effect2	0.0026 (0.0011, 0.0040)	0.0005 (-0.0001, 0.0011)	-0.0166 (-0.0545, 0.0213)
**Turn point of 13 years old**	**20**	**69.5**	**0.7613**
< Turn point, effect1	0.0138 (0.0084, 0.0191)	0.0042 (0.0014, 0.0070)	-0.0573 (-0.1166, 0.0020)
> Turn point, effect2	0.0029 (0.0013, 0.0046)	0.0010 (0.0004, 0.0016)	0.0891 (-0.0194, 0.1977)
**Turn point of 14 years old**	**19.3**	**71.4**	**0.2624**
< Turn point, effect1	0.0313 (0.0231, 0.0396)	0.0109 (0.0075, 0.0142)	-1.1514 (-1.8918, -0.4110)
> Turn point, effect2	0.0031 (0.0016, 0.0047)	0.0005 (-0.0002, 0.0012)	-0.0012 (-0.0540, 0.0516)
**Turn point of 15 years old**	**21.5**	**65.7**	**0.7883**
< Turn point, effect1	0.0197 (0.0144, 0.0251)	0.0188 (0.0065, 0.0310)	-0.0721 (-0.1371, -0.0070)
> Turn point, effect2	0.0035 (0.0017, 0.0052)	0.0016 (0.0009, 0.0022)	0.0579 (-0.0664, 0.1823)
**Turn point of 16 years old**	**23.9**	**84.7**	**0.8027**
< Turn point, effect1	0.0149 (0.0113, 0.0185)	0.0044 (0.0030, 0.0058)	-0.0890 (-0.1594, -0.0186)
> Turn point, effect2	0.0030 (0.0011, 0.0049)	0.0007 (-0.0002, 0.0016)	0.2030 (0.0467, 0.3592)
**Turn point of 17 years old**	**24.5**	**79.7**	**0.2111**
< Turn point, effect1	0.0124 (0.0084, 0.0164)	0.0041 (0.0020, 0.0062)	-4.2717 (-6.1958, -2.3475)
> Turn point, effect2	0.0014 (-0.0007, 0.0034)	0.0004 (-0.0005, 0.0012)	0.0386 (-0.0184, 0.0955)
**Turn point of 18 years old**	**21.8**	**78**	**0.2019**
< Turn point, effect1	0.0145 (0.0081, 0.0209)	0.0029 (0.0005, 0.0053)	1.6428 (0.3547, 2.9309)
> Turn point, effect2	0.0020 (0.0005, 0.0035)	0.0003 (-0.0004, 0.0010)	-0.0144 (-0.0718, 0.0429)
**Turn point of 19 years old**	**33.9**	**117**	**0.3562**
< Turn point, effect1	0.0076 (0.0056, 0.0096)	0.0019 (0.0011, 0.0027)	-0.2356 (-0.5011, 0.0300)
> Turn point, effect2	-0.0039 (-0.0079, 0.0002)	-0.0024 (-0.0052, 0.0004)	0.0186 (-0.0559, 0.0930)

Adjusted for all confounding factors.

The model is not adjusted for the stratification variable itself in the subgroup analysis.

BMI and WC VS total BMD. Left leg fat/lean VS left leg BMD.

### The association between WC and BMD

**Figure 3:**
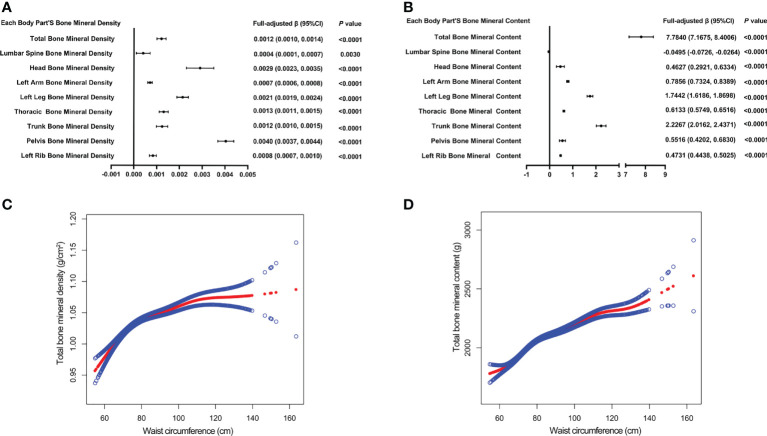
The forest plots of each body part’s bone mineral density or bone mineral content and waist circumference, respectively **(A, B)**. Association of total bone mineral density and bone mineral content with waist circumference **(C, D)**. The solid red line represents the smooth curve fit between variables. Blue bands represent the 95% confidence interval from the fit **(C, D)**. All confounding factors were adjusted.

**Table 4** presents three weighted multiple linear regression models. All variables were adjusted, and there was a statistically significant positive correlation between BMD and WC in all three models. Trend analysis was statistically significant (*P* for trend < 0.05) when BMI was stratified by quartile and the lowest quartile was used as a reference point. The positive associations for total BMD, left leg BMD, and WC remained significant in subgroups stratified by gender, age, standing height, and ethnicity. Likewise, in age-stratified subgroups, this association of WC with lumbar spine BMD was not observed in 12, 13, and 17 - 19 years adolescents. **Figures 3A, B** are forest plots of BMD or BMC and WC for each body part, respectively, and BMD or BMC and WC for each body part have a statistically positive correlation. **Figures 3C, D** are the smooth curve fitting graphs of Total BMD or Total BMC and WC, respectively. When we smooth curve-fit the revised model, there is a saturation effect for total BMD and WC (**Figure 3C**). We also conducted saturation effect model research to determine the WC tipping point and determined that the WC saturation effect value was 70.5 cm. When WC was less than 70.5 cm, BMD increased by 0.0054 g/m^2^ for each unit increase in WC. However, when WC was greater than 70.5 cm, BMD increased by just 0.0010 g/m^2^ for each unit increase in WC. As shown in **Table 3**, the WC saturation effect values for teenagers of different ages were different in the subgroups that were split up by age. Likewise, when we separated the data by gender, we found that both males and females had WC saturation values.

**Table 4:** Association between waist circumference (cm) and bone mineral density (g/cm^2^).

Exposure	Total BMDβ (95% CI)	Lumbar Spine BMD β (95% CI)	Left Leg BMD β (95% CI)
**Stratified by quintiles of Waist circumference (cm)**			
Q1 (54.6-71)	reference	reference	reference
Q2 (71.1-78.2)	0.0195 (0.0120, 0.0270)	0.0221 (0.0119, 0.0322)	0.0284 (0.0196, 0.0373)
Q3 (78.3-88.9)	0.0304 (0.0224, 0.0383)	0.0248 (0.0140, 0.0356)	0.0453 (0.0359, 0.0547)
Q4 (89-163.3)	0.0464 (0.0378, 0.0551)	0.0180 (0.0063, 0.0297)	0.0777 (0.0675, 0.0879)
P for trend	< 0.001	0.048	< 0.001
**Stratified by gender**			
Male	0.0010 (0.0007, 0.0013)	0.0006 (0.0002, 0.0010)	0.0017 (0.0013, 0.0021)
Female	0.0015 (0.0012, 0.0018)	0.0004 (-0.0001, 0.0008)	0.0028 (0.0025, 0.0032)
**Stratified by age (years old)**			
12	0.0006 (0.0002, 0.0011)	0.0002 (-0.0005, 0.0008)	0.0021 (0.0015, 0.0027)
13	0.0012 (0.0007, 0.0017)	0.0004 (-0.0002, 0.0010)	0.0021 (0.0015, 0.0027)
14	0.0011 (0.0006, 0.0017)	0.0011 (0.0003, 0.0018)	0.0018 (0.0011, 0.0025)
15	0.0016 (0.0010, 0.0022)	0.0010 (0.0002, 0.0018)	0.0021 (0.0014, 0.0028)
16	0.0014 (0.0008, 0.0019)	0.0008 (0.0001, 0.0015)	0.0023 (0.0017, 0.0030)
17	0.0014 (0.0008, 0.0020)	0.0008 (-0.0000, 0.0016)	0.0024 (0.0017, 0.0031)
18	0.0004 (-0.0001, 0.0009)	-0.0001 (-0.0008, 0.0006)	0.0015 (0.0010, 0.0021)
19	0.0007 (0.0001, 0.0013)	-0.0004 (-0.0012, 0.0004)	0.0014 (0.0007, 0.0021)
**Stratified by Standing height (cm)**			
Q1 (132.9-160.3)	0.0019 (0.0015, 0.0022)	0.0008 (0.0003, 0.0014)	0.0036 (0.0032, 0.0040)
Q2 (160.4-169)	0.0011 (0.0007, 0.0015)	0.0003 (-0.0002, 0.0008)	0.0019 (0.0015, 0.0024)
Q3 (169.1-190.9)	0.0013 (0.0009, 0.0016)	0.0008 (0.0003, 0.0013)	0.0019 (0.0014, 0.0023)
**Stratified by Race**			
Mexican American	0.0012 (0.0007, 0.0016)	0.0004 (-0.0002, 0.0009)	0.0022 (0.0016, 0.0027)
Other Hispanic	0.0017 (0.0011, 0.0024)	0.0016 (0.0006, 0.0025)	0.0029 (0.0022, 0.0037)
Non-Hispanic White	0.0012 (0.0008, 0.0016)	0.0002 (-0.0004, 0.0008)	0.0022 (0.0017, 0.0027)
Non-Hispanic Black	0.0010 (0.0005, 0.0014)	0.0006 (-0.0000, 0.0012)	0.0017 (0.0012, 0.0022)
Other race	0.0014 (0.0009, 0.0019)	0.0005 (-0.0001, 0.0012)	0.0018 (0.0013, 0.0024)

Adjusted for all confounding factors.

The model is not adjusted for the stratification variable itself in the subgroup analysis.

### Association between fat/lean mass and BMD of corresponding parts of the body

**Figure 4:**
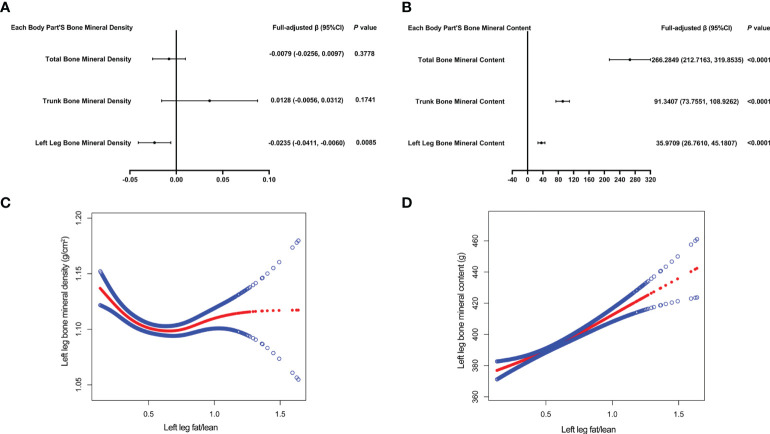
The forest plots of each body part’s bone mineral density or bone mineral content and left leg fat/lean mass, respectively **(A, B)**. Association of left leg bone mineral density and bone mineral content with left leg fat/lean mass **(C, D)**. The solid red line represents the smooth curve fit between variables. Blue bands represent the 95% confidence interval from the fit **(C, D)**. All confounding factors were adjusted.

**Table 5** presents the fully corrected models for total body, trunk, and left leg fat/lean body mass and BMD, respectively. When stratified by quartile for fat/lean mass and using the lowest quartile as the reference point, this trend analysis was statistically significant in the model for only left calf fat-lean mass and BMD (*P* for trend < 0.05), and there is a significant negative correlation. In subgroups stratified by gender, this negative association was only found in male. This negative association was not statistically significant in subgroups stratified by age and standing height. Among the subgroups stratified by race, however, only the other race had a statistically significant negative correlation between left leg fat/lean mass and BMD. **Figures 4A, B** are forest plots of BMD or BMC and fat/lean mass for each body part, respectively. There is a statistically significant negative correlation between left leg BMD and fat/lean mass, and BMC for each body part is associated with fat There was a significant positive correlation with lean body mass. **Figures 4C, D** are fitted with smooth curves drawn in the revised model, there is a saturation effect for left leg fat/lean mass and left leg BMD. The saturation effect value of left leg fat/lean mass was calculated to be 0.2994 using a saturation effect model analysis to determine the turning point for left leg fat/lean mass. When the fat/lean mass ratio of the left leg is smaller than 0.2994, the BMD drops by 0.3185g/m^2^ for each unit of left leg fat/lean mass. When left leg fat/lean mass is more than 0.2994, the BMD drops by 0.0096 g/m^2^ for every unit of increase in left leg fat/lean mass. **Table 3** shows that a turning point of left leg fat/lean mass was only found in teenagers aged 14 to 17 years. And when stratified by gender, we found a saturated value of left leg fat/lean mass in males.

**Table 5:** Association between fat/lean mass and bone mineral density (g/cm^2^) of corresponding parts of the body.

Exposure	Total, β (95% CI)	Trunk, β (95% CI)	Left Leg, β (95% CI)
**Stratified by quintiles of fat/lean mass**			
Lowest quartiles	reference	reference	reference
2nd	-0.0100 (-0.0180, -0.0021)	-0.0034 (-0.0117, 0.0049)	-0.0165 (-0.0260, -0.0070)
3rd	-0.0157 (-0.0243, -0.0071)	-0.0040 (-0.0130, 0.0049)	-0.0154 (-0.0260, -0.0047)
4th	-0.0092 (-0.0189, 0.0006)	0.0021 (-0.0080, 0.0122)	-0.0185 (-0.0302, -0.0069)
*P* for trend	0.053	0.683	0.009
**Stratified by gender**			
Male	-0.0440 (-0.0706, -0.0174)	-0.0306 (-0.0600, -0.0013)	-0.0493 (-0.0756, -0.0230)
Female	0.0289 (0.0047, 0.0531)	0.0510 (0.0276, 0.0745)	0.0174 (-0.0066, 0.0415)
**Stratified by age (years old)**			
12	-0.0500 (-0.0875, -0.0126)	-0.0279 (-0.0682, 0.0123)	-0.0241 (-0.0604, 0.0123)
13	0.0039 (-0.0378, 0.0456)	0.0157 (-0.0283, 0.0596)	-0.0144 (-0.0569, 0.0282)
14	0.0100 (-0.0392, 0.0593)	0.0383 (-0.0116, 0.0882)	-0.0242 (-0.0752, 0.0268)
15	-0.0040 (-0.0581, 0.0501)	0.0216 (-0.0324, 0.0756)	-0.0394 (-0.0925, 0.0137)
16	0.0394 (-0.0178, 0.0966)	0.1002 (0.0434, 0.1570)	-0.0246 (-0.0821, 0.0330)
17	0.0240 (-0.0341, 0.0820)	0.0084 (-0.0498, 0.0665)	0.0183 (-0.0391, 0.0756)
18	-0.0438 (-0.0990, 0.0115)	-0.0358 (-0.0947, 0.0231)	0.0016 (-0.0547, 0.0578)
19	0.0066 (-0.0577, 0.0710)	0.0193 (-0.0474, 0.0859)	-0.0100 (-0.0771, 0.0571)
**Stratified by Standing height (cm)**			
Q1 (132.9-160.3)	0.0120 (-0.0156, 0.0396)	0.0379 (0.0099, 0.0659)	0.0004 (-0.0270, 0.0278)
Q2 (160.4-169)	-0.0059 (-0.0365, 0.0247)	0.0197 (-0.0109, 0.0503)	-0.0304 (-0.0612, 0.0003)
Q3 (169.1-190.9)	-0.0096 (-0.0459, 0.0267)	-0.0048 (-0.0446, 0.0350)	-0.0192 (-0.0565, 0.0181)
**Stratified by Race**			
Mexican American	-0.0229 (-0.0617, 0.0158)	0.0049 (-0.0344, 0.0443)	-0.0271 (-0.0669, 0.0127)
Other Hispanic	0.0122 (-0.0436, 0.0680)	0.0527 (-0.0042, 0.1096)	-0.0052 (-0.0610, 0.0505)
Non-Hispanic White	-0.0125 (-0.0469, 0.0220)	0.0082 (-0.0279, 0.0442)	-0.0298 (-0.0633, 0.0037)
Non-Hispanic Black	0.0016 (-0.0358, 0.0391)	0.0095 (-0.0304, 0.0493)	-0.0054 (-0.0431, 0.0324)
Other race	-0.0155 (-0.0584, 0.0275)	0.0098 (-0.0348, 0.0545)	-0.0447 (-0.0875, -0.0020)

Adjusted for all confounding factors.

The model is not adjusted for the stratification variable itself in the subgroup analysis.

## Discussion

Statistics from the NHANES were used to analyze the connection between obesity status and total BMD in teens ages 12 to 19. In this cross-sectional study of adolescents, a significantly positive connection was observed between BMI, WC, and total BMD. These conclusions appear to be consistent with previous studies (16–20). However, the association between left leg fat/lean mass and left leg BMD was negative. Moreover, through fitting smooth curve and saturation effect model analysis, it was shown that the saturation effect values of BMI, WC, and left leg fat/lean mass were 22 kg/m^2^, 70.5 cm, and 0.2994, respectively. As BMI and WC surpassed this effective value, the degree of the rise in total BMD diminished. The magnitude of the increase in total BMD decreased when BMI and WC exceeded this effect size. After the fat/lean mass of the left leg was lower than the effect value, the BMD of the left leg decreased accordingly. As previously stated (21, 22)when the body fat rate is lower than 33%, the body fat content is positively correlated with bone density, reducing the risk of fractures to a certain extent, but when the body fat rate is higher than 33%, body fat content in most skeletal areas is inversely correlated with BMD. Our findings are in line with these conclusions.

Obesity status and OP have emerged as major public health issues that are receiving increased attention. Obesity status and OP, however, they are controversial subjects. According to a Tartu cross-sectional study (23), obese boys showed greater BMD values than their normal-weight classmates. In a similar study, the researchers evaluated the BMD of female teenagers and separated them into two groups: the fat group and the normal group. Significantly increased BMD was found in the obese group compared to the normal group (24). A meta-analysis and systematic review include 27 studies found that people who are overweight or obese have much higher BMD than people who are a healthy weight (25). Nevertheless, there is research that has produced results that are contradictory (26, 27). The WC is frequently used as an indicator of abdominal obesity. The linear regression analysis revealed a markedly adverse connection between WC and BMD in a sample of 271 adolescents, including those with and without metabolic syndrome (MS). Among the components of MS, the connection between increased WC and decreased BMD is the strongest (28). Although obesity benefits BMD, numerous studies show that having a high BMI significantly raises one’s personal risk of developing type 2 diabetes, pre-diabetes, dyslipidemia, non-alcoholic fatty liver disease, and heart conditions, the underpinning mechanisms involved include inflammation, oxidative stress, and mitochondrial dysfunction (29–35). Faienza et al. (36) believe that these mechanisms of obesity can increase the possibility of osteoporosis and brittle fractures.

Presently, the mechanism between Obesity and OP is uncertain. There were multiple mechanisms may exist. to begin with, excess body fat deposition and significantly high obesity result in increased static mechanical compliance (37, 38), which causes static mechanical pressures on bone and a series of changes in bone structure. Secondly, obesity increases the number and metabolic rate of adipocytes in the bone marrow. The bone marrow has cells called bone mesenchymal stem cells (BMSCs). These cells can change into osteoblasts and adipocytes. Obesity can stimulate the development of BMSCs into adipocytes, a result of the increase of bone marrow adipocytes as well as a reduction of bone marrow osteoblasts (39). The inappropriate buildup of bone marrow adipocytes in the skeletal portion will lead to an imbalance in osteocyte activity and a reduction in bone turnover. It can easily result in the start of OP at an earlier age (40). Thirdly, obesity contributes to inflammation. The proliferation of adipocytes in the microenvironment of bone marrow will hasten the release of pro-inflammatory and immunoregulatory substances. In addition to accelerating the production and activation of osteoclasts, these inflammatory substances also limit the release of osteoprotegerin, diminish the differentiation of osteoblasts, and induce osteoclasts (41). Fourth, people who are overweight or obese have a higher synthesis and release of endocrine hormones, including estrogen, insulin, leptin, etc. These hormones inhibit bone resorption and bone remodeling and thus exert a beneficial influence on BMD (42–46). Fifth, obesity alters genes connected to obesity. For example, the Pro10 allele of tumor necrosis factor-1 (47), the leptin gene (48), and the fourth receptor gene for melanocorticoid (49). Studies show that these gene mutations make people more likely to be overweight and hurt their bones.

Nevertheless, we must equally acknowledge that our findings have many drawbacks. Because of the study’s cross-sectional methodology, there could not be a causative connection established between lower BMD and being overweight or obese. Secondly, we could not gather full-scale data of participants regarding their living habits, eating habits, prescription data, bone metabolism indicators, and endocrine hormones that regulate bone metabolism. Our findings suggest that there are statistically significant differences between men and women in indicators such as moderate exercise, blood biochemical markers, and body composition, which may be partly explained by shifts in hormone levels during puberty, as well as differences in exercise patterns, venues, etc. It may lead to differences in sun exposure time, exposed parts, etc., thereby affecting calcium and phosphorus metabolism. Additionally, we were unable to gather menstrual histories from female participants. Finally, we could not identify participants with a history of fractures, osteoarthritis, premature infants, anorexia, etc. Previous studies have reported that these factors affect bone health during adolescence (50, 51). In addition, this study investigated the association between obesity status and BMD, which, although most important for bone health, does not 100% represent bone health, as Longhi et al. and Vibha et al. both found an increased risk of bone fractures in the extremities of adolescents with excess obesity compared with average weight, which may be related to reduced bone strength due to reduced cross-sectional and cortical areas of the skeleton (27, 52).

Our study included a sizable and geographically representative sample pool to draw from. Similar to what was reported in previous studies, our weighted multiple linear regression analysis demonstrated that obesity status was favorably associated with enhanced BMD. When we conducted smooth curve fitting in a model which adjusted for all the variables, we found that the effect sizes between BMI, WC, fat/lean mass, and BMD, respectively, were saturated. According to our findings, the total BMD reached saturation when the BMI of adolescents was 22kg/cm^2^ and the WC was 70.5cm. At the same time, when fat/lean body mass < 0.2994, left leg BMD and fat/lean mass were significantly negatively correlated, and we noticed that there was a correlation with age. As a result, we recommend that adolescents keep their BMI, WC, and fat/lean mass close to the saturation effect value in order to allows adolescents to have better bone mass development but not excessive obesity.

## Data availability statement

Publicly available datasets were analyzed in this study. The survey data can be found here: https://www.cdc.gov/nchs/nhanes/.

## Ethics statement

The studies involving human participants were reviewed and approved by The NHANES required every individual who took part in the survey to sign an informed consent form, which was then reviewed and authorized by the National Center for Health Statistics Ethics Review Board. The data can now be accessed by the general public following privacy-preserving. It is already possible to transform data into a form that can be analyzed. All statistics would be used for data analysis and all studies will be done in compliance with applicable laws and standards provided we comply with the study’s data usage guidelines. Written informed consent to participate in this study was provided by the participants’ legal guardian/next of kin.

## Author contributions

G-XW and Z-BF contributed equally to this study. All authors contributed to the article and approved the submitted version.

## Funding

H-LL was supported by the Shenzhen Municipal Science and Technology Innovation Council (No. JCYJ20170817094838619). The funder had no role in study design, data collection and analysis, decision to publish, or preparation of the manuscript. S-FC was supported by the National Natural Science Foundation of China (NO. 82104759). The funder had no role in study design, data collection and analysis, decision to publish, or preparation of the manuscript. S-FC was supported by the Natural Science Foundation of Guangdong Provincial (NO. 2019A1515110108). The funder had no role in study design, data collection and analysis, decision to publish, or preparation of the manuscript. S-FC was supported by the Shenzhen Municipal Science and Technology Innovation Council (No. JCYJ20180302173821841). The funder had no role in study design, data collection and analysis, decision to publish, or preparation of the manuscript.

## Conflict of interest

The authors declare that the research was conducted in the absence of any commercial or financial relationships that could be construed as a potential conflict of interest.

## Publisher’s note

All claims expressed in this article are solely those of the authors and do not necessarily represent those of their affiliated organizations, or those of the publisher, the editors and the reviewers. Any product that may be evaluated in this article, or claim that may be made by its manufacturer, is not guaranteed or endorsed by the publisher.
